# The Pathology of Seborrhœa

**Published:** 1878-05

**Authors:** 


### Selections.

---

The Pathology of Seborrhœa.—Some time ago, while investigating the pathology of certain affections of the skin in which the epithelium plays a prominent part, I had occasion to examine microscopically the product of disease thrown off in the various forms of seborrhœa, as found in different parts of the body. Somewhat to my surprise I found the epithelium to present a different character in some cases of the affection known as seborrhœa sicca capitis from that shown in the other forms of seborrhœa. In a brief account of these investigations published last year I expressed a doubt suggested by the results of my microscopic examinations, as to the propriety of calling this affection seborrhœa. "It does not," I said, "consist essentially in an excessive flow of abnormal sebum, but in the exfoliation of the epidermis, (from the stratum corneum) mingled indeed with sebaceous matter to a greater extent than is the case in the other squamous affections of the scalp, but nevertheless presenting epidermis as its principal pathological product." I found myself unable at that time to follow up this statement with any further details, but I propose in the present paper to take the subject up once more, and while noting some facts in the pathology of seborrhœa in general, to attempt in particular the demonstration of the essentially non-seborrhœic character of the seborrhœa sicca capitis of Hebra and others, at least in some of its forms. In order to do this I shall first offer some remarks upon the histology of the sebaceous glands with their product. I shall then give the result of examination in those affections which are beyond question seborrhœic in character. Having presented the sebaceous glands in their normal and pathological condition, I shall examine the product of disease in the affection particularly in question, and by a comparison of this with the normal type of seborrhœic disease, and also by referenc to the histological facts stated, shall endeavor to establish my proposition. Finally, I

shall adduce the evidence of other writers who have arrived at the same conclusions with myself, through studying the affection from a somewhat different standpoint.

*Histology of the Sebaceous Glands.*—The sebaceous glands may be regarded as involutions of the skin, or, in some cases, of the hair sacs. That all the layers of the skin however do not take part in this involution will appear, if we picture to ourselves a normal gland seen in section, as described by the histologists. We observe that the outer portion of the gland is composed of an external coat of connective tissue, continued in the case of free glands from corium, in other cases continued from the hair sac. (Kolliker.) This may he called the gland sac, and its interior, with the exception of a small central cavity, is filled with epithelial cells, directly continuous with those of the stratum malpighii, of which the most external that lie in contact with the gland sac, resemble the deeper cells of the mucous layer, except only that the nucleus is more distinctly visible. Those that are situated more internally first become filled with small fat molecules, and then with larger fat drops that surround and conceal the nucleus and cause the cells to increase in size. The cavity of the sebaceous gland is occupied by an amphorous mass of fatty matter, and the debris of numerous cells. (Biesiadecki.) The stratum corneum does not take any part in the formation of the gland itself. As it dips down into the funnel-shaped opening of the sebaceous sac it appears to become more and more attenuated, and either ends at the neck of the gland proper, or perhaps lines a portion of the neck. This absence of the stratum corneum in the structure of the sebaceous gland would be a matter of indifference in reference to the present subject, if the same views prevailed as formerly regarding the genesis of the horny cells from those of the stratum mucosum. Kolliker says: "The formation of the cutaneous sebaceous matter resembles in many respects that of the cuticle. The young, easily soluble cells at the bottom of the glandular follicles may be compared to the malpighian cells of the epidermis, and the less soluble ones of the secretion filled with fat to the horny plates." But the recent researches of Langerhans prove that the stratum corneum is absolutely separated from the stratum mucosum by the stratum lucidum of Oehl and Schron, and that the latter alone forms the germinal layer for the horny cells of the cuticle. We cannot therefore regard the cells of the sebaceous secretion as homologous with those of the horny layer, and if in any affection supposed to involve the sebaceous glands, we find under the microscope cells from the stratum corneum, we are driven to suppose, either that these are adventitious accompaniments of the seborrhœal affection, or, if the horny cells are in great excess, that the sebaceous material has been poured out to some extent, in connection with an affection essentially epidermoidal in character.

*Pathology of the Sebaceous Glands.*—Proceeding now from the histology of the sebaceous gland to its pathology, let us examine the product of secretion in those inflammatory conditions in which this is abnormal in quality or quantity or both. If we express the plug-like mass of comedo from the gland containing it, and cutting off the outer third of its length, examine the remainder under the microscope, we should expect to find all the elements of the sebaceous secretion characteristically displayed. I have done this with the following result: a number of comedo plugs prepared as above were digested for some days in ether, and the solid matter remaining was stained with aniline, and examined under the microscope. The major part of the field was occupied by cells, with some glandular debris. These cells were colored darkly by the aniline, with the exception of a large vacuole in the centre which remained quite light. The surrounding cell contents showed advanced fatty degeneration, being composed of fat granules and globules. No cells resembling those of the horny layer were observed.

In seborrhœa oleosa we should also expect to find the true glandular secretion, only poured out in excess, and with the addition of a certain number of horny cells derived from the general surface, or perhaps from the funnel-shaped apertures of the glands. Having scraped the oily product lightly from the surface of the nose in a well-marked case of seborrhœa oleosa, I placed a portion upon a glass slide, and after treating it with aniline examined it microscopically. The field as in the case of comedo was largely occupied with deeply stained epithelial cells, their contents showing advanced fatty degeneration. The nucleus which was usually small and light-colored, was occasionally shrunken and surrounded by a bright areola. The cell contents consisted entirely of granules or globules of fat. In some cases the outline was dim or jagged as if the cell was about breaking down into fat globules and debris. There was much granular matter in the field with *a few* unmistakeable horny cells.

I suppose that seborrhœa oleosa is a precisely analogous affection to comedo, the difference between them being that in the latter, the chemical constitution of the sebum is altered, while in s. oleosa this is unchanged, being merely increased in quantity. Here then we have in two undoubtedly seborrhœic affections no cells resembling the horny cells of the epidermis, excepting that in the case of seborrhœa oleosa a few are encountered evidently adventitious in their occurrence.

There is one variety of seborrhœa sicca which is essentially the same disease with those just mentioned. This is found in its most characteristic form on the chest and back in the shape of nummular or annular patches made up of a reddish base, surmounted by yellowish-brown, fatty, pellicle-like scales, occasionally massed together to form a greasy coating. I have examined the product of disease in this variety of seborrhœa with the following result: The disease from which the specimens were taken was composod of patches and rings of yellowish-brown oily scales, of a pearly, greasy lustre, and having a doughy feeling when pressed between the finger and thumb. They contained so much oily material as to leave large stains in the bit of paper on which they were lying. Treated with aniline and water the cells colored pretty well, though they were somewhat difficult to stain, owing to the repulsive action of the commingled oil. Under the microscope the cell contents were found to be decidedly granular. In some cells minute oil globules could be observed; the cell outline was frequently indistinct. Many cells contained a nucleus which in some cases appeared shrunken, and was contained within a vacuole; in others the place of the nucleus was occupied by a vacuole alone.

Thus far all is plain. The product of the disease is essentially the same in comedo, seborrhœa oleosa and seborrhœa sicca corporis. I have reason to believe that certain forms of seborrhœa sicca capitis, those in which the eruption is similar to that described as dry seborrhœa of the body, present the same microscopic appearances. I have not, however, had an opportunity of examining these. It is when we come to examine that variety of seborrhœa which is characterized by the formation of fine, dry, powdery, or pearly white scales, constituting a branny disquamation of the scalp, that we find decided differences in the microscopic appearances presented. The following notes of an examination of a case of this kind will show just what these differences are: The scales taken from the scalp of a young girl, who had suffered a long time with severe "dandruff," were macerated for some weeks in ether, and then stained with carmine. Under the microscope the cells were sharp in outline, without distinctly granular contents; no sign of fatty degeneration. Most of the cells contained large distinct nuclei, much lighter in color than the protoplasm.

It is very evident, I think, that we have here a different product from that remarked in the first three observations. There we had the typical product of the sebaceous gland, the granular cell, sometimes complete, again breaking down, and finally broken into granules and globules of oily matter. Here we find nothing of the kind. The cells are those of the horny layer, and although differing in appearance from the epithelial cells of the horny layer thrown off in eczema squamosum and psoriasis, yet evidently belong to the same stratum. We have in this form of disease, to which the term of seborrhœa can, I think, no longer be properly applied, something intermediate, pathologically speaking, between seborrhœa sicca and eczema and psoriasis. For this the term pityriasis or pityriasis simplex might properly be employed. In times past this term was used to denote a large number of affections having a desquamation of the epidermis as their chief feature, but being distinct in other respects. In the natural reaction from this confused nomonclature, we have come of late to confine the term to the disease known as pityriasis subra alone. The field, therefore, remains clear for the introduction of the title pityriasis simplex, to denote an affection whose pathology and clinical aspects alike exemplify the idea conveyed by the term.

At the time the examinations were made, which led me to the conclusions above stated, I was unaware that similar views, as to the existence of a true pityriasis, had been reached by others. I have since ascertained that such views have been sustained, in opposition to that of Hebra, by Pincus and Piffard. Pincus, after stating Hebra's views, says, that while admitting the value of the latter's observations, he cannot subscribe to the conclusions drawn from them. Pincus collected the scales from the scalp in a number of cases of ordinary dandruff, dried them, and after weighing them carefully, digested them in ether, weighed the sediment, and examined it microscopically. The result of seven such estimations showed an average loss of three-fifths the entire mass. The remaining two-fifths were epidermis. On two occasions Pincus had an opportunity of observing pityriasis capitas complicated by seborrhœa. The seborrhœa appeared, and ran an acute and severe course, so that the old pityriasis alone remained at the end of fourteen days in one case, and at the end of three and a half weeks in the other case. Having examined the scales in one case every third day, and in the other every second day, during the course of the disease, he found that when the disease was at its height, the proportion of pure epidermis in the scales was one-ninth to one-eleventh of the entire mass. During the latter part of the disease, as it was disappearing, the old proportion of two to five was again observed. Pincus goes on to say, that where the seborrhœic matter poured out is almost fluid, and the skin looks as if covered with oil, the proportion by weight of epidermis must be much smaller. He inclines to the plan of calling by the designation seborrhœa the disease heretofore known by that name, while the affection just alluded to should be called pityriasis, with the understanding that this designation does not involve a denial of the existence of something more than a mere increased desquamation of epidermis.

Piffard, speaking independently of Pincus, and making no reference to his statements, arrives at a similar conclusion. He says: "Upon microscopic examination, the scales (of pityriasis) will be found to be constituted chiefly of horny cells, with a varying, sometimes very slight, amount of entangled sebum." That there is an affection, which may with propriety be called seborrhœa sicca, Piffard admits. For this disease he prefers the name acne sebacæ. Piffard gives a picture of the microscopic cells in pityriasis, which resemble precisely those seen and noted by me.

Finally, the facts above stated may be formulated as follows:

1. The sebaceous secretion is derived from fatty metamorphosis of the enchyma cells of the sebaceous glands. These cells are homologous with those of the stratum mucosum of the skin. They have nothing in common with the cells of the horny layer.

2. Seborrhœa is a disease of the sebaceous glands, characterized by the pouring out of an increased quantity of sebum, more or less altered in chemical and physical composition. In comedo and seborrhœa sicca, properly so called, the secretion is condensed to a fatty consistency, while in seborrhœa oleosa it remains in an oily state. In each of these affections, however, microscopic examination shows epithelial cells in a state of more or less complete fatty degeneration, and breaking down into granular debris. Horny cells are only found adventitiously.

3. Certain forms of disease, heretofore commonly classed as seborrhœa sicca, should properly be removed from the category of diseases of the sebaceous glands, since the pathological product in these cases is not sebum, but epithelium, from the horny layer of the skin. Any sebum which may be present is a mere accompaniment of the epithelial product. For these cases the designation pityriasis, or pityriasis simplex, would seem appropriate.

